# The Current State of Remote Physiotherapy in Finland: Cross-sectional Web-Based Questionnaire Study

**DOI:** 10.2196/35569

**Published:** 2022-06-07

**Authors:** Thomas Hellstén, Jari Arokoski, Tuulikki Sjögren, Anna-Maija Jäppinen, Jyrki Kettunen

**Affiliations:** 1 Faculty of Medicine University of Helsinki Helsinki Finland; 2 School of Engineering, Culture and Wellbeing Arcada University of Applied Sciences Helsinki Finland; 3 Department of Physical and Rehabilitation Medicine Helsinki University Hospital Helsinki Finland; 4 Faculty of Sport and Health Sciences University of Jyväskylä Jyväskylä Finland; 5 Department of Internal Medicine and Rehabilitation Helsinki University Hospital Helsinki Finland; 6 Graduate School and Research Arcada University of Applied Sciences Helsinki Finland

**Keywords:** COVID-19, remote physiotherapy, COVID-19 pandemic, current state, suitability in disease groups, competence of physiotherapist

## Abstract

**Background:**

The ongoing COVID-19 pandemic has required social, health, and rehabilitation organizations to implement remote physiotherapy (RP) as a part of physiotherapists’ daily practice. RP may improve access to physiotherapy as it delivers physiotherapy services to rehabilitees through information and communications technology. Even if RP has already been introduced in this century, physiotherapists’ opinion, amount of use, and form in daily practice have not been studied extensively.

**Objective:**

This study aims to investigate physiotherapists’ opinions of the current state of RP in Finland.

**Methods:**

A quantitative, cross-sectional, web-based questionnaire was sent to working-aged members of the Finnish Association of Physiotherapists (n=5905) in March 2021 and to physiotherapists in a private physiotherapy organization (n=620) in May 2021. The questionnaire included questions on the suitability of RP in different diseases and the current state and implementation of RP in work among physiotherapists.

**Results:**

Of the 6525 physiotherapists, a total of 9.9% (n=662; n=504, 76.1% female; mean age 46.1, SD 12 years) answered the questionnaire. The mean suitability “score” (0=not suitable at all to 10=fully suitable) of RP in different disease groups varied from 3.3 (neurological diseases) to 6.1 (lung diseases). Between early 2020 (ie, just before the COVID-19 pandemic) and spring 2021, the proportion of physiotherapists who used RP increased from 33.8% (21/62) to 75.4% (46/61; *P*<.001) in the public sector and from 19.7% (42/213) to 76.6% (163/213; *P*<.001) in the private sector. However, only 11.7% (32/274) of physiotherapists reported that they spent >20% of their practice time for RP in 2021. The real-time method was the most common RP method in both groups (public sector 46/66, 69.7% vs private sector 157/219, 71.7%; *P*=.47). The three most commonly used technical equipments were computers/tablets (229/290, 79%), smartphones (149/290, 51.4%), and phones (voice call 51/290, 17.6%). The proportion of physiotherapists who used computers/tablets in RP was higher in the private sector than in the public sector (183/221, 82.8% vs 46/68, 67.6%; *P*=.01). In contrast, a higher proportion of physiotherapists in the public sector than in the private sector used phones (18/68, 26.5% vs 33/221, 14.9%; *P*=.04).

**Conclusions:**

During the COVID-19 pandemic, physiotherapists increased their use of RP in their everyday practice, although practice time in RP was still low. When planning RP for rehabilitees, it should be considered that the suitability of RP in different diseases seems to vary in the opinion of physiotherapists. Furthermore, our results brought up important new information for developing social, health, and rehabilitation education for information and communications technologies.

## Introduction

Providing easy and equal access to physiotherapy services is a significant challenge due to the aging population; increasing prevalence of chronic diseases; and the concentration of health, rehabilitation, and social services to urban areas [1,2]. Physiotherapy is a profession with expertise in health, movement, mobility, and function [3,4]. Remote physiotherapy (RP), or alternatively telerehabilitation (this term was introduced in the late 90s in the scientific literature [5]), offers a means to improve the availability of physiotherapy as it delivers physiotherapy services to rehabilitees through information and communications technology (ICT) [5-11]. RP opens the possibility for new work tasks and new approaches for physiotherapists in examination, implementation, and follow-up, which affect their professional role [3]. While RP can involve direct online communication with a physiotherapist, such that the rehabilitee and the physiotherapist are physically in two different places, RP can also mean a digital application used in physiotherapy that provides automatic feedback and support for the rehabilitee [12]. In this paper, we use the term RP to describe how conventional physiotherapy is delivered remotely using ICT. The term rehabilitee is defined as a patient, client, customer, or group, and the real-time method describes direct online communication between the rehabilitee and physiotherapist.

The COVID-19 pandemic has required health care organizations to implement RP as a part of physiotherapists’ daily practice [13]. RP has allowed physiotherapists to continue their daily clinical practice during the pandemic for those rehabilitees that need physiotherapy but are unable to visit a hospital or clinic. RP has also supported social distancing to reduce the spread of COVID-19 [13,14] and has been implemented in COVID-19 physiotherapy [15-17], although we have not focused on this in our study.

RP may be as effective as conventional physiotherapy in some disease groups, such as musculoskeletal diseases [2,18-20], heart and lung diseases [9,21], or neurological diseases [22]. Moreover, a major advantage over conventional physiotherapy is that the rehabilitee does not need to travel for RP, thus saving time and travel costs. Another positive consequence is that the rehabilitee can decide for themselves when to perform their therapeutic exercise, and it is easier to implement the exercise into their daily activity [12,20,23].

Despite the advantages of RP, physiotherapy is still typically practiced in person. There are several barriers that preclude the wider use of RP. These include the physiotherapist’s competence in using technical equipment and resistance to RP; technical investment costs; and the age, degree of education, and computer literacy of the rehabilitee [24]. Environmental space and infrastructural challenges such as bandwidth capacity are other barriers to RP for both rehabilitees and physiotherapists [25].

There is some evidence that the COVID-19 pandemic has increased the use of RP in Switzerland [26] and in Kuwait [27]. However, our knowledge of the current state of RP in Finland is limited. Therefore, we conducted a study to determine how appropriate RP is for different disease groups, the proportion of practice time spent on RP before and during the COVID-19 pandemic, which method and what technology physiotherapists use on RP, and the difference between public and private sector use of RP.

## Methods

### Study Design

We used a quantitative, cross-sectional, web-based questionnaire study to answer the research questions. Physiotherapists responded to the questionnaire anonymously. This study adhered to the CHERRIES (Checklist for Reporting Results of Internet E-Surveys) [28] and The STROBE (Strengthening the Reporting of Observational Studies in Epidemiology) Statement [29].

The term RP was defined as a physiotherapy intervention that includes remote technology such as telephones, smartphones, computers, tablets, activity trackers, computer vision (CV), artificial intelligence (AI), virtual reality (VR), or robotics such that the physiotherapist is physically in a different place than the rehabilitee [7]. The terms real-time and not-tied-to-time methods were defined as follows: a real-time method is online communication between rehabilitee and physiotherapist; a method not tied to time means remote technology used in physiotherapy that provides automatic feedback and support for the rehabilitee [12].

The Finnish health care system consists of two complementary sectors that receive public funding, the public and private sector. There are substantial differences between these systems, such as scope of services provided, user fees, and waiting times. There are also differences in financing mechanisms. The public sector is financed based on taxes and the National Health Insurance (NHI); the private sector is partly (one-third) financed by NHI [30]. Therefore, we analyzed these sectors separate in our study. Although there are two different sectors, every rehabilitee has the right to good and equal quality health care and rehabilitation.

### Subjects

We recruited physiotherapists of working age from the Finnish Association of Physiotherapists (n=5905) and from a private physiotherapy organization (n=620). A questionnaire was mailed to physiotherapists in March (Finnish Association of Physiotherapists) and May 2021 (private physiotherapy organization) via an information letter that included an electronic link to the questionnaire. The questionnaire had a 5-week deadline. Two reminders were sent during this period; the first reminder was sent after 1 week and the second reminder 2 weeks after the first.

### The Questionnaire

A questionnaire was constructed that included items that were based on previous literature in the field [10,23,31-33] and on the opinions of the research teams and coworkers (that included experts such as medical doctors, physiotherapists, clinical specialists, researchers, and lecturers) working in a university hospital, city health station, university, university of applied sciences, and physiotherapy association. The questionnaire was piloted by 28 physiotherapists from different physiotherapy fields and geographical locations in Finland. In the pilot phase, we asked for feedback on the questionnaire, such as unclear questions and suggestions for corrections. Word choices were changed, and two questions were changed from compulsory to optional.

The questionnaire included 32 questions (31 closed and 1 open question). To study suitability of RP in different diseases and patients with pain, we used an 11-point numeric scale (0=not suitable at all, 10=fully suitable). While most of the patients with chronic pain are patients with musculoskeletal disorder [34], we inserted them into the category “musculoskeletal diseases.” The numeric rating scale was chosen as it is well understood and used in physiotherapy [35]. Other questions included were “how much of your practice time have you spent on RP in the month before the survey,” “how much of your practice time have you spent on RP just before the COVID-19 pandemic (early 2020),” “do you use real-time methods or methods not tied to time in RP,” and “which of the following technology solutions do you use weekly in RP.”

### Statistical Methods

Statistical analyses were performed with SPSS (Version 27.0; IBM Corp). Frequency distributions, percentages, and means are given as descriptive statistics. Chi-square statistics and Student *t* test were applied to calculate statistical differences between the public and private groups. *P*<.05 (2-tailed) was considered as a statistically significant threshold.

### Ethical Considerations

The study was granted ethical approval by the research ethics committee of the Faculty of Medicine at University of Helsinki in February 2021 (registration number 3/2021).

## Results

Of the 6525 physiotherapists, a total of 9.9% (n=662) answered the questionnaire. Physiotherapy students and physiotherapists that were retired, lecturers, or researchers were excluded; the final study group included 579 (8.9%) physiotherapists. Of these 579 physiotherapists, 482 (83.2%) were females (mean age 49.3 SD 11.9 years), and 97 (16.8%) were males (mean age 46.2, SD 12.2 years). Of the physiotherapists, 423 (73.1%) worked in the private sector and 152 (26.3%) in the public sector; in addition to these, 3 did not answer this specific question, and 1 could not be classified to either group.

Physiotherapists in the public and private sector typically had extensive work experience. Almost four-fifths (440/579, 76%) had over 10 years of experience; there was no difference in work experience between the physiotherapists in these two sectors. However, the proportion of physiotherapists who reported that they do not have work experience in RP was higher in the public sector than in the private sector. Detailed characteristics of the physiotherapists are shown in Table 1.

There were minimal differences when the mean suitability “score” (0=not suitable at all to 10=fully suitable) of RP in different connected disease groups between public and private sectors were compared. However, the mean suitability “score” of lung diseases (*P*=.02) and in musculoskeletal diseases (*P*=.01) was higher in the public than private sector. The mean suitability “score” of RP in different diseases varied from 2.1 (memory disorder) to 6.6 (hip or knee osteoarthritis, asthma). Only 9.7% (40/411) considered asthma and 8.2% (37/452) considered hip or knee osteoarthritis as fully suitable (score 10) for RP; 32.2% (134/416) considered RP not suitable at all (score 0) for rehabilitees with memory disorder (Table 2).

Three-quarters of all physiotherapists reported that they did not spend any of their practice time in RP before the COVID-19 pandemic in early 2020. The proportion of such physiotherapists was higher in the private sector than in the public sector (171/213, 80.3%; vs 41/62, 66.1%; *P*=.03). Only a few physiotherapists spent more than 20% of their practice time for RP (Table 3).

Between early 2020 and spring 2021, the proportion of physiotherapists who used RP increased from 33.8% (21/62) to 75.4% (46/61; *P*<.001) in the public sector and from 19.7% (42/213) to 76.6% (163/213; *P*<.001) in the private sector. The proportion of physiotherapists who did not use RP in 2021 was only 24.6% (15/61) in the public sector and 23.5% (50/213) in the private sector with no statistically significant group difference (*P*=.86). However, the proportion of physiotherapists who used over 20% of their practice time on RP was still minimal. Detailed results are shown in Table 3.

When studying the methods and equipment used in individual RP, the real-time method was the most common method in the public (46/66, 69.7%) and the private (157/219, 71.7%) sector. In contrast, only a few physiotherapists used the method not tied to time (Table 4); a corresponding result was seen in group RP (data not shown). In the total group, the three most used technical equipment were computers/tablets (229/290, 79%), smartphones (149/290, 51.4%), and phones (51.290, 17.6%; voice call). The proportion of physiotherapists who used computers/tablets in RP was higher in the private sector than in the public sector (183/221, 82.8% vs 46/68, 67.6%; *P*=.01). However, a higher proportion of physiotherapists in the public sector than in the private sector used phones (18/68, 26.5% vs 33/221, 14.9%; *P*=.04). Other equipment such as VR, CV, or AI were rarely used (Table 4).

**Table 1:** Characteristics of the study physiotherapists.

		Total group (n=579)	Public sector (n= 152)	Private sector (n=423)	*P* value	
**Age (years), mean (SD)**						
	Total	48.8 (11.9)	48.6 (11.9)	49.0 (11.9)	.73^a^	
	Female	49.3 (11.9)	49.3 (11.9)	49.3 (11.7)	.93^a^	
	Male	46.2 (12.2)	42.3 (10.3)	47.3 (12.3)	.15^a^	
Time from physiotherapy degree (years), mean (SD)		22.3 (12.6)	21.4 (12.5)	22.7 (12.5)	.27^a^	
**Work experience in physiotherapy, n (%)**						.47^b^
	<1 year	18 (3.1)	7 (4.6)	10 (2.4)		
	≥1 year and <5 years	65 (11.2)	17 (11.2)	47 (11.1)		
	≥5 years and <10 years	56 (9.7)	12 (7.9)	43 (10.2)		
	≥10 years	440 (76.0)	116 (76.3)	323 (76.4)		
**Work experience in remote physiotherapy, n (%)**						<.001^b^
	No experience	210 (36.3)	77 (50.7)	130 (30.7)		
	<1 year	209 (36.1)	26 (30.3)	162 (38.3)		
	1 year to 2 years	135 (23.3)	26 (17.1)	109 (25.8)		
	>2 to 4 years	13 (2.2)	1 (0.7)	12 (2.8)		
	>4 years	12 (2.1)	2 (1.3)	10 (2.4)		

^a^*P* values are based on Student *t* test.

^b^*P* values are based on chi-square test.

**Table 2:** Suitability score of remote physiotherapy in different disease groups^a^.

Connected disease groups and subgroups			Total group, mean (SD)		Public sector, mean (SD)		Private sector, mean (SD)		Mean difference (95% CI)		*P* value^b^
**Lung diseases**			6.1 (2.4)		6.5 (2.1)		5.9 (2.5)		0.6 (0.1 to 1.1)		.02
	Asthma	6.6 (2.5)		6.8 (2.3)		6.5 (2.5)		0.3 (–0.3 to 0.8)		.31	
	COPD^c^	5.6 (2.6)		6.2 (2.2)		5.4 (2.7)		0.8 (0.3 to 1.3)		.003	
**Musculoskeletal diseases**			5.7 (2.2)		6.1 (1.9)		5.6 (2.3)		0.6 (0.1 to 1.0)		.01
	Knee and hip osteoarthritis	6.6 (2.5)		7.2 (2.1)		6.4 (2.6)		0.8 (0.3 to 1.2)		.001	
	Low back pain	5.9 (2.6)		5.9 (2.5)		5.9 (2.6)		0.0 (–0.5 to 0.5)		.98	
	Repetitive strain injury of the hand and forearm	5.9 (2.8)		6.5 (2.7)		5.6 (2.8)		0.9 (0.3 to 1.5)		.002	
	Tendon disorder of the shoulder	5.8 (2.7)		6.0 (2.6)		5.7 (2.7)		0.4 (–0.2 to 0.9)		.19	
	Rheumatoid arthritis	5.7 (2.5)		6.1 (2.3)		5.5 (2.6)		0.6 (0.1 to 1.2)		.02	
	Pain patient	5.2 (2.7)		5.3 (2.6)		5.1 (2.7)		0.2 (–0.4 to 0.8)		.50	
	Neck pain	4.8 (2.7)		4.7 (2.6)		4.8 (2.7)		–0.1 (–0.7 to 0.5)		.75	
**Psychiatric diseases**			4.9 (2.7)		5.3 (2.6)		4.7 (2.7)		0.6 (0.0 to 1.2)		.06
	Anxiety disorder	5.2 (3.0)		5.6 (3.1)		5.0 (3.0)		0.6 (0.0 to 1.3)		.045	
	Depression	5.0 (2.9)		5.3 (2.8)		4.8 (2.9)		0.5 (–0.2 to 1.1)		.15	
	Personality disorder	4.7 (2.9)		4.9 (2.9)		4.6 (2.9)		0.4 (–0.2 to 1.0)		.23	
**Neurological diseases**			3.3 (2.1)		3.3 (1.9)		3.3 (2.2)		0.1 (–0.5 to 0.4)		.81
	Multiple sclerosis	4.4 (2.6)		4.3 (2.4)		4.4 (2.7)		–0.1 (–0.6 to 0.5)		.85	
	Parkinson disease	4.0 (2.6)		4.1 (2.5)		4.0 (2.6)		0.1 (–0.5 to 0.7)		.69	
	Cerebral infarction (eg, stroke)	3.3 (2.6)		3.1 (2.4)		3.4 (2.6)		–0.2 (–0.8 to 0.3)		.45	
	Spinal cord injury	3.2 (2.7)		2.9 (2.3)		3.3 (2.8)		–0.5 (–1.1 to 0.1)		.09	
	Brain injury	3.2 (2.5)		2.9 (2.4)		3.2 (2.6)		–0.3 (–0.8 to 0.2)		.25	
	Memory disorder	2.1 (2.2)		2.3 (2.3)		2.0 (2.2)		0.3 (–0.2 to 0.8)		.21	
**Other**											
	Heart disease/failure	5.8 (2.7)		6.1 (2.5)		5.7 (2.8)		0.4 (–0.1 to 1.0)		.14	
	Cancer	5.2 (2.8)		5.3 (2.7)		5.2 (2.8)		0.1 (–0.5 to 0.7)		.75	
	Multimorbid patient	3.6 (2.6)		3.7 (2.6)		3.5 (2.7)		0.2 (–0.4 to 0.8)		.46	

^a^Suitability score (0=not suitable at all to 10=fully suitable).

^b^*P* values are based on Student *t* test.

^c^COPD: chronic obstructive pulmonary disease.

**Table 3:** Proportion of physiotherapists who used remote physiotherapy before (early 2020) and during the COVID-19 pandemic (spring 2021).

Proportion of practice time (%)		Total group, n (%)	Public sector, n (%)	Private sector, n (%)	*P* value^a^	
**Before COVID-19 pandemic**						.03
	0	212 (76.8)	41 (66.1)	171 (80.3)		
	1-20	60 (21.7)	19 (30.6)	40 (18.8)		
	>20	4 (1.4)	2 (3.2)	2 (0.9)		
**During the COVID-19 pandemic**						.20
	0	65 (23.7)	15 (24.6)	50 (23.5)		
	1-20	177 (64.6)	35 (57.4)	142 (66.7)		
	>20	32 (11.7)	11 (18.0)	21 (9.9)		

^a^*P* values are based on chi-square tests.

**Table 4:** Methods and equipment used in remote physiotherapy on a weekly basis.

			Total group, n (%)		Public sector, n (%)		Private sector, n (%)		*P* value^a^	
**Method**										.47
	Real-time method	203 (71.0)		46 (69.7)		157 (71.7)				
	Method not tied to time	11 (3.8)		1 (1.5)		10 (4.6)				
	Real-time method and method not tied to time	25 (8.7)		5 (7.6)		20 (9.1)				
**Equipment**										
	Computer/tablet	229 (79.0)		46 (67.6)		183 (82.8)		.01		
	Smartphone	149 (51.4)		33 (48.5)		116 (52.5)		.58		
	Phone	51 (17.6)		18 (26.5)		33 (14.9)		.04		
	Activity tracker^b^	18 (6.2)		3 (4.4)		15 (6.8)		.58		
	Others^c^	10 (1.7)		1 (1.5)		9 (4.1)		.76		

^a^*P* values are based on chi-square tests.

^b^For example, pedometer and accelerometer.

^c^Exergame, television application, virtual reality, computer vision, artificial intelligence, robotics, smart textile, or augmented reality.

## Discussion

### Principal Findings

This study sought to investigate physiotherapists’ opinion on the current state of RP in Finland. While the ongoing COVID-19 pandemic has increased the use of RP in everyday practice, practice time for RP was still minimal, as just 1 in 10 used >20% of practice time to conduct RP. The suitability of RP varied across different disease groups. According to the physiotherapists, RP is better suited for rehabilitees with lung, heart, or musculoskeletal diseases than for rehabilitees with neurological diseases. RP was most commonly performed with a computer/tablet or a smartphone and with real-time methods. Less than 2% of physiotherapists used other technological equipment (eg, VR, AI, or CV).

The COVID-19 pandemic has led to the rapid adoption of RP by hospitals and clinics. RP has enabled physiotherapists to continue to provide therapy to rehabilitees during the pandemic, prevent further transmission of the virus, and decrease the burden of the health system during this period [14,36]. Rapid implementation of RP was also observed in our study; however, we did not assess the use of RP with rehabilitators due to the COVID-19 pandemic. Although still low, the number of physiotherapists who reported use of RP in their practice during the study period increased. One explanation for the rapid implementation of RP at the beginning of the COVID-19 pandemic may be that the Social Insurance Institution of Finland temporarily restricted conventional physiotherapy, and clinics and hospitals were thus required to use RP. Prior to the pandemic, RP was used more in the public sector than in the private sector, which may be due to strategic decisions in the public organizations. On the other hand, private sector companies are usually smaller and more dynamic, and this may partly explain the rapid implementation of RP in the private sector. Data security and protection systems are usually more complex in the public sector, which may have also affected implementation of RP.

In the private sector, 4 in 5 physiotherapists did not use RP at all prior to the COVID-19 pandemic, in contrast to 2 in 3 in the public sector. During the study period, the proportion of physiotherapists who reported that they do not use RP has decreased to slightly over 20% in both sectors. This increased use of RP observed in our study is consistent with the findings of Rausch et al [26] who observed that RP increased from 4.9% (prior to the COVID-19 pandemic) to 44.6% (during the COVID-19 pandemic). In their study, physiotherapists aged <45 years used RP more than the older ones [23]. A corresponding relationship between age and RP use was not observed in our study (data not shown).

Previous studies indicate that RP is comparable to conventional physiotherapy for rehabilitees with stroke [22,37], hip and knee osteoarthritis [38], chronic respiratory disease [11], and multiple sclerosis [22]. In our study, the suitability “score” of RP seemed to be higher among certain diseases (eg, asthma or knee and hip osteoarthritis) in which verbal communication, such as guidance and advice, is a key element. Similarly, Rausch et al [26] concluded that RP is used the most in guidance and advice for rehabilitees. In contrast, RP seems to be poorly suitable for rehabilitees with memory disorders and spinal cord injuries. However, the current disease state should be considered when planning physiotherapy. It may be that RP is suitable in the early phase of, for example, neurological diseases, when hands-on therapy is not essential. Overall, knowledge on RP as an alternative or as a part of conventional physiotherapy in different diseases is still limited.

Physiotherapy has traditionally been a hands-on profession, and thereby physiotherapist may find it challenging to reach the standard of conventional physiotherapy with RP. RP may require changes in work routines and skills, as well as a greater workload and changes in interaction with rehabilitees [39]. RP cannot be used as replacement for the necessary contact between the rehabilitee and the physiotherapist [21] and should not replace conventional physiotherapy [26,32]. Further, barriers for RP that have been presented are demands in communication through a screen, lack of physical contact with rehabilitee, short of appropriate rehabilitation equipment in rehabilitees environment, digital literacy [32,40], and appropriate financial compensation [26]. In some countries, insurance companies hesitate to cover RP; however, it is not an issue in Finland where physiotherapists can decide what method to use, conventional physiotherapy or RP. It should be noted that real-time methods, which are the most used form of RP, still offer real-time contact between the rehabilitee and physiotherapist even if the medium is digital. In our study, 71% (203/286) of the physiotherapists reported having used real-time methods, 3.8% (11/286) methods not tied to time, and 8.7% (25/286) both methods. RP may offer opportunities to work more effectively with methods that are not tied to time, but the use of these methods is rare. However, the advantages and disadvantages of such methods should be tested in high-quality interventional studies.

A computer or tablet was the most chosen communication medium. This is comparable with previous findings that reported that physiotherapists preferred real-time methods with video technologies over other mediums [26,36,41,42]. Moreover, the possibilities that the technology provides to the physiotherapy process, rather than the method or technology itself, are important. Rehabilitees who are not interested in or are unfamiliar with the technology require more conventional physiotherapy than enthusiastic rehabilitees who see advantages on the use of technology and feel that RP could offer sufficient support [8].

In this study, almost three-quarters of the physiotherapists had no experience or had <1 year experience of RP, which can affect the use of RP. A previous study revealed that work experience is associated with the perception of how convenient RP is in clinical practice [41]. The willingness to use RP among physiotherapists has been reported to be high [27]. For easy implementation of RP in everyday practice, attention should be paid to not only professional education and skill training [26,32] but also common technical problems [43]. On the other hand, hardware and software costs are decreasing, ICT speeds are increasing, and the technology is continuously developing, which collectively have a positive effect on the use of RP [44]. The use of RP is still rare, but appropriate technology coupled with professional education in RP for undergraduate and recently graduated physiotherapists allows for an increase in the implementation of RP.

### Strengths and Limitations

A strength of this study is the number of physiotherapists (n=662) who answered the survey, even if only a total of 9.9% (662/6525) answered. The physiotherapists were recruited from all municipalities in Finland and included physiotherapists with short and long clinical experience. Our physiotherapists could somehow be generalizable to the broader Finnish physiotherapy workforce, where 82% of employed physiotherapists are female with a mean age of 44.8 years, and the physiotherapists have relatively long clinical experience.

Our study also had some limitations. Our survey data were collected in Finland, and our findings may not be generalizable to other countries where physiotherapists may have more experience in RP and with a different health care system. The proportion of physiotherapists in the private sector who answered the questionnaire was higher than the corresponding proportion in the overall Finnish physiotherapy workforce in the private sector. Some of the physiotherapists in the private physiotherapy organization are also members of the Finnish Association of Physiotherapists and had the possibility to respond twice to the questionnaire. To avoid such an overlap, we recommended in the information letter not to respond twice. Furthermore, we do not know the reasons for overrepresentation of physiotherapists from the private sector in our study, but we analyzed the private and public sector separately.

Further, one limitation of our study may be nonparticipation bias. We recruited the study physiotherapists from the Finnish Association of Physiotherapists and from a private physiotherapy organization, but we had to collect the data anonymously. Therefore, it was not possible to analyze whether responders were significantly different from nonresponders and how these possible differences influenced the results of the study. Lastly, the use of a scientifically unvalidated questionnaire can be seen as a limitation. However, the questionnaire was based on consensus in a broad expert group, essential literature in the field, and was pilot-tested.

### Conclusions

Based on our results, the suitability of RP for different diseases varies. During the COVID-19 pandemic, physiotherapists increased use of RP in their clinical practice, but use is still rare. To conduct RP, physiotherapists use a computer/tablet or a smartphone and use a real-time method. Other technological equipment and methods are used infrequently. These results may help physiotherapists and organizations in planning and implementing RP in everyday work and in the development of physiotherapy education of ICT.

## Abbreviations

AI: artificial intelligence
CHERRIES: Checklist for Reporting Results of Internet E-Surveys
CV: computer vision
ICT: information and communications technology
NHI: National Health Insurance
RP: remote physiotherapy
STROBE: Strengthening the Reporting of Observational Studies in Epidemiology
VR: virtual reality
